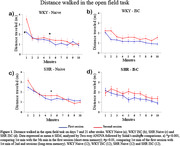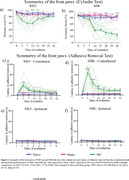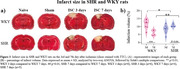# Influence of Systemic Arterial Hypertension on Memory and Motor Recovery after Permanent Focal Cerebral Ischemia in Spontaneously Hypertensive Rats

**DOI:** 10.1002/alz.092309

**Published:** 2025-01-03

**Authors:** Francieli Rohden, João Victor Matos e Moreira, Luis Pedro Bernardi, Nicolly Paz, Thomas Hugentobler Schlickmann, Lucas Uglione Da Ros, Eduardo R. Zimmer, Diogo O. Souza

**Affiliations:** ^1^ Federal University of Rio Grande do Sul (UFRGS), Porto Alegre, RS Brazil; ^2^ Federal University of Rio Grande do Sul, Porto Alegre, RS Brazil; ^3^ Universidade Federal do Rio Grande do Sul, Porto Alegre, Rio Grande do Sul Brazil; ^4^ Universidade Federal Do Rio Grade Do Sul, Porto Alegre, Rio Grande do Sul Brazil; ^5^ McGill University, Montreal, QC Canada; ^6^ Brain Institute of Rio Grande do Sul ‐ Pontifícia Universidade Católica do Rio Grande do Sul, Porto Alegre, Rio Grande do Sul Brazil

## Abstract

**Background:**

Systemic Arterial Hypertension (SAH), distinguished by a persistent elevation of blood pressure, emerges as a risk factor for stroke and Alzheimer’s Disease (AD). Additionally, recent evidence suggests that stroke may adversely affect memory, potentially playing a role in the development of AD. This study aimed to investigate the influence of permanent focal ischemic stroke on memory, as well as on sensorimotor function (asymmetry of the front paws) and cerebral infarct size in adult male spontaneously hypertensive rats (SHR), compared to normotensive Wistar Kyoto (WKY) rats.

**Method:**

We assessed the stroke effects on short‐ and long‐term memory through the open field task (7 and 21 days after stroke), on sensorimotor functions through the cylinder test and adhesive removal test (over 42 days after stroke), and on infarction volume through 2,3,5‐Triphenyltetrazolium chloride staining (3 and 7 days after stroke).

**Result:**

Short‐term memory was observed in both naive WKY and naive SHR rats, while SHR naive animals did not exhibit long‐term memory. Stroke disrupted short‐term memory in both WKY and SHR rats and long‐term memory in WKY rats (Fig. 01). In regards to the sensorimotor function, (cylinder test and adhesive removal) WKY rats totally recovered from the stroke‐induced asymmetry in the front paws, while SHR rats did not (Fig. 02). Concerning infarction volume, WKY rats showed decreased infarction volume from the third to the seventh day after stroke, while SHR rats did not exhibit this reduction (Fig. 03).

**Conclusion:**

These results emphasize the impact of hypertension on memory, as well as on motor outcomes and brain infarct size following stroke, potentially contributing to the risk for AD.